# Survival from breast cancer among South Asian and non-South Asian women resident in South East England

**DOI:** 10.1038/sj.bjc.6601097

**Published:** 2003-07-29

**Authors:** I dos Santos Silva, P Mangtani, B L De Stavola, J Bell, M Quinn, D Mayer

**Affiliations:** 1 Department of Epidemiology and Population Health, London School of Hygiene and Tropical Medicine, Keppel Street, London WC1E 7HT, UK; 2 Thames Cancer Registry, 1st floor, Capital House, Weston Street, London SE1 3QD, UK; 3 National Cancer Intelligence Centre, Office for National Statistics, 1 Drummond Gate, London SW1 V 2QQ, UK

**Keywords:** breast cancer, survival, ethnicity, migrants, South Asians

## Abstract

Ethnic differences in breast cancer survival have been observed in the USA but have not been examined in Britain. We aimed to investigate such differences between South Asian (i.e. those with family roots in the Indian subcontinent) and non-South Asian (essentially British-native) women in England. Primary breast cancer cases incident in 1986 –1993 and resident in South East England were ascertained through the Thames Cancer and Registry and followed up to the end of 1997. Cases of South Asian ethnicity were identified on the basis of their names by using a previously validated computer algorithm. A total of 1037 South Asian and 50 201 non-South Asian breast cancer cases were included in the analysis; 30% of the South Asian (*n*=312) and 44% (*n*=22 201) of the non-South Asian cases died during follow-up. South Asian cases had a higher relative survival than non-South Asians throughout the follow-up period. The 10-year relative survival rates were 72.6% (95% confidence interval: 69.0, 75.9%) and 65.2% (64.5, 65.8%) for South Asians and non-South Asians, respectively. The excess mortality rates experienced by South Asians were 82% (72, 94%) of those experienced by non-South Asians (*P*=0.004). The magnitude of this effect was slightly reduced with adjustment for differences in age at diagnosis, but was strengthened with further adjustment for differences in stage at presentation and socioeconomic deprivation (excess mortality rates in South Asians relative to non-South Asians=72% (63, 82%), *P*<0.001). These findings indicate that the higher survival from breast cancer in the first 10 years after diagnosis among South Asian was not due to differences in age at diagnosis, socioeconomic deprivation or disease stage at presentation.

Breast cancer is the most commonly occurring malignant neoplasm among women in England and Wales accounting for about 30% of all female malignancies and about 20% of all female cancer deaths (Swerdlow *et al*, 2001). Survival from this cancer has improved steadily over time (Coleman *et al*, 1999) due to increasing breast cancer awareness, earlier diagnosis, increasing use of effective adjuvant therapy and, more recently, the introduction of the NHS Breast Screening Programme.

Large socioeconomic differences in breast cancer survival have been observed in England and Wales (Coleman *et al*, 1999). Ethnic differences in survival from breast cancer have also been reported in the USA (Young *et al*, 1981; Richardson *et al*, 1992; Eley *et al*, 1994; Hsu *et al*, 1997; Hunter, 2000) but have never been examined in Britain. South Asian ethnic populations (i.e. those with family roots in the Indian subcontinent irrespective of their place of birth) are one of the largest minority groups in Britain representing 2.7% (almost 1.5 million) of the total population (Office of Population Census and Surveys (OPCS) and General Register Office (GRO) for Scotland, 1993). Although South Asian women living in Britain have lower breast cancer incidence than British-native women (Winter *et al*, 1999), breast cancer is still the commonest female cancer among South Asian migrants and there have been concerns that their survival from this tumour may be poorer due to later presentation (Hoare, 1996; Selby, 1996). In this paper, we use data from the Thames Cancer Registry, which covers one of the areas in Britain with the largest concentration of South Asians (OPCS and GRO for Scotland, 1993), to assess differences in survival from breast cancer between South Asian and non-South Asian women, the latter group comprising essentially British-native women (OPCS and GRO for Scotland, 1993).

## MATERIALS AND METHODS

### Data sources

The Thames Cancer Registry is a population-based cancer registry that covers South East England, with a female population of about 7 million, 3. 8% of whom are of South Asian ethnic origin (OPCS and GRO for Scotland, 1993). This registry collects data on cancer patients from a number of sources including hospital records, pathology laboratories and death certificates (Thames Cancer Registry, 1995). Patient follow-up is carried out passively through linkage to the National Health Service Central Register, which provides information on deaths, emigrations and losses to follow-up. We extracted data from the Thames Cancer Registry on all primary breast cancer cases incident from 1986 to 1993. Cases incident after 1993 were excluded to ensure completeness of registration at the time of data extraction and a reasonable length of follow-up. Nonresidents in the South East and patients with primaries in other organs (except nonmelanoma skin cancer) were excluded. Patients with more than one primary in the breast were included only if the first was diagnosed during 1986–1993 and their survival time calculated from this date.

Data on ethnicity were not available in the registry files. A recently developed computer algorithm, SANGRA, was therefore used to identify South Asian individuals by matching the name of each study subject in the registry files to the South Asian names contained in its directories. The validity of this algorithm has been shown to be high (sensitivity ranging from 89 to 96% and specificity from 94 to 98%) (Nanchahal *et al*, 2001). SANGRA also classifies South Asian names according to their religious origin as Hindu, Moslem or Sikh with fairly high validity (sensitivity ranging from 84 to 98% and specificity from 85 to 98%). To further improve SANGRA's specificity and thus minimise the number of false positives, all names identified by this algorithm as being South Asians were visually inspected and the data reanalysed after the exclusion of names known to be common to other ethnic groups. A woman's socioeconomic circumstances at the time of breast cancer diagnosis were ascertained by the Carstairs index, an area-based measure of socioeconomic deprivation (Carstairs and Morris, 1989). Data on four 1991 Census variables (percentages of household overcrowding, car ownership, male unemployment and social class IV or V) were first combined to give a composite score of deprivation for each enumeration district in Britain (Carstairs and Morris, 1989) and the resulting distribution categorised into fifths, ranging from 1 (‘affluent’) to 5 (‘deprived’). The full postcode of the usual residence of each woman at the time of diagnosis was then linked to the corresponding enumeration district and the patient assigned to one of these five deprivation categories. Only a small proportion of hospital records included staging of the cancer at diagnosis (24% in early 1990; Chouillet *et al* (1994)), but registry staff used data from pathology and operation reports, and other sources, to classify tumours into four stages: 1=local; 2=extension to surrounding tissues; 3=local node involvement; and 4=presence of metastases (Thames Cancer Registry, 1995).

### Statistical methods

The follow-up time of each woman was calculated as the number of days between date of diagnosis and date of death, emigration, loss to follow-up or 31 December 1997 (the last date for which follow-up data were regarded as complete at the time of data extraction), whichever occurred first. To adjust for mortality from causes other than breast cancer, relative survival rates (RSRs), expressed as percentages, were calculated using the Estève *et al* (1990) method. For a given time since diagnosis, the RSR is the ratio of the survival probability observed in the group of cancer patients being studied to the survival probability that would have been expected if they had experienced the same mortality rates as a reference population. This approach is recommended when information on cause of death is not known or unreliable (as with population-based cancer registry data) because the assumption of proportionality of the mortality rates (or hazards), implicit in the most commonly used survival models (e.g. Cox or Poisson), is rarely satisfied (Estève *et al*, 1990). England and Wales period- and age-specific female life tables were used to compute expected survival probabilities for both South Asians and non-South Asians as ethnic-specific life tables are not available. Separate life tables for North and South Thames, but not for the whole South East, have been published (Coleman *et al*, 1999), and since they produced results similar to those obtained with the national one, only the latter are presented here. To assess separately how RSRs in South Asians and non-South Asians were influenced by prognostic factors, and to compare them directly while accounting for the potential confounding effects of these factors, a generalisation of the Estève *et al* method (suggested by Dickman *et al*, 2003, in press) was used to compute *excess mortality rate ratios*. These represent the mortality rates experienced by breast cancer patients with a certain characteristic relative to those experienced by the baseline group, once the reference population mortality is taken into account (see the Appendix). The significance of the effects, linear trends and possible interactions were assessed using likelihood ratio tests (Clayton and Hills, 1993).

## RESULTS

A total of 59 816 female primary breast cancer cases incident in 1986–1993 were eligible for the study, 1. 9% (1123) of whom were identified by SANGRA as being of South Asian ethnic origin. In total, 86 (7.7%) South Asian and 8414 (14.3%) non-South Asian cases were excluded because the date of death was known but not the date of diagnosis (death certificate only cases), and a further 78 cases because of invalid or missing ages at diagnosis, birth or death. Thus, 1037 South Asian and 50 201 non-South Asian breast cancer cases were included in the analyses, of whom 312 (30%) South Asians and 22 201 (44%) non-South Asian cases died during follow-up. The median follow-up times of the breast cancer cases who did not die during the study period were similar for South Asians (7.2 years (25th – 75th centiles: 5.6 – 9.2) and non-South Asians (7.0 years (5.5 – 8.9)).

**Figure 1:**
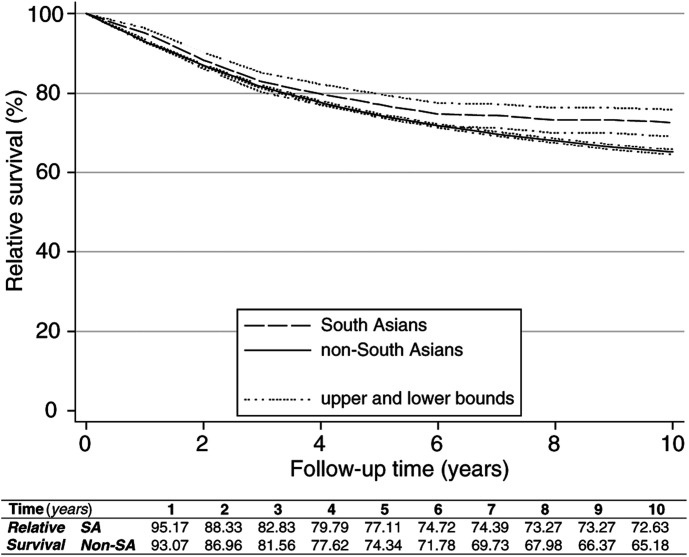
Relative survival rates and 95% confidence bounds by ethnicity.

South Asian cases had higher relative survival than non-South Asians (Figure 1

). The 5-year RSR was 77.1% (95% confidence interval=74.2, 79.3%) for South Asians and 74.3% (73.9, 74.8%) for non-South Asians. The corresponding 10-year figures were 72.6% (69.0, 75.9%) and 65.2% (64.5, 65.8%), respectively.

South Asian breast cancer cases were younger at diagnosis than non-South Asian cases, a reflection of underlying differences in the age structure of these populations, but less likely to live in affluent areas (Table 1

**Table 1:** Excess^a^ mortality rate ratios (RRs) and 95% confidence intervals (CI) for the available prognostic factors by ethnicity

	**Univariate analysis**								
	**South Asian women (n=1037)**				**Non-South Asian women (n=50201)**				
**Risk factor**	***n***	**(%)**	**RR**	**(95% CI)**	***n***	**(%)**	**RR**	**(95% CI)**	**Heterogeneity**^b^
Age (years)									
<45	483	(47)	1		11 035	(22)	1		
45–64	419	(40)	0.80	(0.61, 1.07)	17 397	(35)	0.94	(0.90, 0.99)	
⩾65	135	(13)	1.13	(0.73, 1.75)	21 769	(43)	1.40	(1.33, 1.46)	
									
*Linear trend*^c^				*P*=0.63				*P*<0.001	*P*=0.56
									
Period of diagnosis									
1986–1989	556	(54)	1		30 324	(60)	1		
1990–1993	481	(46)	0.72	(0.55, 0.94)	19 877	(40)	0.71	(0.68, 0.74)	
									
*Heterogeneity*^d^				*P*=0.02				*P*<0.001	*P*=0.53
									
Stage									
1 (local)	358	(35)	1		23 346	(46)	1		
2	132	(13)	2.23	(1.37, 3.62)	4710	(9)	1.88	(1.74, 2.03)	
3	240	(23)	3.09	(2.07, 4.60)	11 002	(22)	2.95	(2.80, 3.11)	
4 (metastases)	99	(10)	8.38	(5.54, 12.68)	4500	(9)	12.46	(11.80, 13.15)	
NK	208	(20)	1.54	(0.95, 2.51)	6643	(13)	1.70	(1.59, 1.83)	
									
*Linear trend*^e^				*P*<0.001				*P*<0.001	*P*=0.06
									
Socioeconomic Deprivation									
1 (affluent)	145	(14)	1		13 151	(26)	1		
2	150	(14)	1.39	(0.81, 2.38)	11 875	(24)	1.10	(1.04, 1.16)	
3	205	(20)	1.29	(0.77, 2.16)	10 311	(21)	1.22	(1.15, 1.29)	
4	234	(23)	1.53	(0.94, 2.50)	8716	(17)	1.35	(1.28, 1.43)	
5 (deprived)	291	(28)	1.71	(1.07, 2.74)	5734	(11)	1.46	(1.37, 1.56)	
NK	12	(1)	2.35	(0.83, 6.65)	414	(1)	1.18	(0.95, 1.47)	
									
*Linear trend*^e^				*P*=0.02				*P*<0.001	*P*=0.86

NK=not known.

a The excess mortality RRs for the categories of the risk factors in the table were computed from relative survival models, each including one of the risk factors under study and fitted separately in South Asian and non-South Asian women. The RRs represent the estimated effects that each separate factor has on excess mortality rates.

b Significance of the likelihood ratio test for heterogeneity between South Asian and non-South Asian women.

c Significance of the likelihood ratio test for linear trend in the category-specific rates.

d Significance of the likelihood ratio test for heterogeneity between the category-specific rates.

e Significance of the likelihood ratio test for linear trend computed excluding the NK category.

). There were no ethnic differences in the proportion of tumours presenting late (stages 3–4). In each ethnic group, excess mortality rates over the follow-up period were highest in cases aged over 64 years at diagnosis (only significantly in non-South Asians), those diagnosed before 1990, those who presented later, and those who lived in the most deprived areas. Although the proportion of tumours of unknown stage was greater for South Asians, in each ethnic group excess mortality rates for women with unknown tumour stage were similar to those with early stage. There was no evidence of an interaction between any of these prognostic factors and ethnicity, except for borderline evidence that the effect of late stage might be stronger in non-South Asians (Table 1).

The excess mortality rates experienced by South Asian cases were statistically significantly lower than those experienced by non-South Asians (Table 2

**Table 2:** Excess^a^ mortality rate ratios (RRs) and 95% confidence intervals (CI) for South Asian ethnicity adjusted for other prognostic factors

	**Effect of ethnicity^b^**		
**Adjusted for**	**RR**	**(95% CI)**	***P*-value**
None	0.82	(0.72, 0.94)	0.004
Age	0.88	(0.78, 1.01)	0.07
Period of diagnosis	0.84	(0.74, 0.96)	0.009
Stage	0.70	(0.61, 0.80)	<0.001
Socioeconomic deprivation	0.77	(0.67, 0.87)	<0.001
Age, period of diagnosis^c^, stage and socioeconomic deprivation	0.72	(0.63, 0.82)	<0.001

a The excess RRs for ethnicity are computed from relative survival models which included, when applicable, other covariates. The estimated RRs represent the estimated effects of being of South Asian ethnic origin on the excess mortality rates.

b Ethnicity is defined as being South Asian *vs* being non-South Asian.

c Including interaction terms between age and period of diagnosis.

). Simultaneous adjustment for all the available prognostic factors, including interaction terms between age and period at diagnosis (which identified the age-specific effect of the NHS Breast Screening Programme introduced in 1989), strengthened the association between ethnicity and breast cancer survival, with excess mortality rates among South Asian cases being only 72% (63, 83%) of those among non-South Asians. The survival advantage observed for South Asians overall was present in each religious group (excess mortality rates relative to those in non-South Asian women were 0.84% (0.70, 1.01%) for Hindus (*n*=458), 0.61% (0.49, 0.76%) for Moslems (*n*=430), and 0.70% (0.49, 0.98%) for Sikhs and others combined (*n*=149)). There was some evidence, based on small numbers, that Moslem women had a slightly better survival (*P*=0.08 for test for heterogeneity). Ten percent of the women classified by SANGRA as South Asian had, on visual inspection, names that were common to other ethnic groups but their exclusion from the analysis did not affect the results (excess mortality rate ratio for ethnicity adjusting for all the available prognostic factors=0.70 (0.61, 0.81)). Similarly, visual inspection of names for a random sample of 1000 women not identified by SANGRA as South Asians showed that only two (0.2%) were potentially missed by the algorithm.

## DISCUSSION

To our knowledge this is the first study to have examined ethnic differences in survival from breast cancer and, indeed, from any cancer in adulthood, in Britain. Studies in the USA, however, have highlighted marked ethnic variations in breast cancer survival (Young *et al*, 1981; Richardson *et al*, 1992; Eley *et al*, 1994; Hsu *et al*, 1997; Hunter, 2000). Reasons for these variations are unclear. Among ethnic minorities presentation tends to occur at a later stage of the disease (Richardson *et al*, 1992; Eley *et al*, 1994; Hsu *et al*, 1997; Hunter, 2000), but this does not seem to fully explain their poorer survival (Young *et al*, 1981; Richardson *et al*, 1992; Eley *et al*, 1994; Hsu *et al*, 1997). Our study provides no evidence that South Asian breast cancer cases in South East England presented later or had worse survival than non-South Asians cases.

Use of name analysis to identify people of South Asian ethnicity has been shown to be valid as most South Asian names are distinct (Nanchahal *et al*, 2001). Despite the very high specificity of SANGRA, there was still potential for some non-South Asians to have been misclassified as South Asians as some names are common to other ethnic groups. Unpublished data from the 1991 Census show that Moslems from other population groups (e.g. North Africans and Arabs) are essentially concentrated in Greater London, but even here, they would represent only 7% of the total number of females who could potentially be regarded as South Asians by SANGRA. Moreover, exclusion of names common to other ethnic groups did not affect the present findings. Name-analysis would fail to identify the few South Asian women in England who had Anglicised or Christian names or those who changed their surnames on marriage to partners of a different ethnic group (but data from the 1991 Census shows that less than 5% of South Asian women in England and Wales married to men belonging to a different ethnic group (Coleman and Salt, 1996)).

The Carstairs index has been shown, in predominantly British-native populations, to be more closely associated with mortality levels than individual-based measures such as social class (Carstairs and Morris, 1989), but it is not known how accurately it captures standards of living among South Asians. A higher proportion of non-South Asians in the present study lived in affluent rather than deprived areas reflecting both the relative affluence of the South East within Britain and the higher incidence of breast cancer in women of high socioeconomic status. In contrast, the majority of South Asian cases lived in relatively deprived areas. Levels of household overcrowding, no access to car, male unemployment and low social class are known to be greater in South Asians than in non-South Asians (OPCS and GRO for Scotland, 1993). Household overcrowding, however, may, in part, reflect the joint nature of some South Asian families rather than their level of affluence.

The unavailability of ethnic-specific life tables could have biased the results if mortality from causes other than breast cancer were different for South Asians and non-South Asians. Mortality data by ethnicity are not available in England and Wales, but all-cause mortality at ages 20–69 years for the period 1989–1992 was similar for women born in the Indian subcontinent and the whole England and Wales female population irrespective of their place of birth (SMR=100; 95% CI=97, 103) (Wild and McKeigue, 1997), the latter comprising essentially non-South Asian women. Although analyses by country of birth are not equivalent to analyses by ethnicity, the large majority of South Asian breast cancer cases in the Thames Cancer Registry have been found to be first-generation migrants (dos Santos Silva *et al*, 2002). National deprivation-specific life tables are available (Coleman *et al*, 1999), but were not used to produce the results shown here because there is no information on ethnic differences in mortality by deprivation to assess whether they would have been appropriate. However, reanalyses using deprivation-specific life tables produced identical results to those shown here (excess mortality rate ratio=0.72 (0.63, 0.83; *P*<0.001) for South Asians *vs* non-South Asians, after adjustment for prognostic factors).

Exclusion of death certificate only cases might have biased the results as such cases tend to be associated with shorter survival (Pollock and Vickers, 1995). As the proportion of these cases was lower in South Asians than in non-South Asians, their exclusion would have led, if anything, to an underestimation of the true survival advantage of the South Asian cases. Apparent survival in South Asians would have been increased if some were lost to follow-up because they returned to their country of origin and died there, but information on migration might not have reached the NHS Central Register and, hence, the Thames Cancer Registry at the time of data extraction. To examine this possibility, the status of the South Asian cases still alive on 31 December 1997 was checked again in 2000. Those who had not died in the meantime were still registered with a general practitioner, implying that they had not migrated out of the country during our follow-up period.

The observed breast cancer survival rates among South Asian women living in England were higher than those reported by population-based cancer registries in India (5-year relative survival rates in India ranged from 42 to 55%) (Gajalakshmi *et al*, 1997). Although these comparisons are somewhat biased by the lack of comparability of the age-structure of the patient populations and the healthy migrant effect, they are likely to reflect true differences in survival, possibly due to higher breast cancer awareness, availability of early detection activities, and access to diagnostic and treatment facilities in England.

South Asian women in England are known to be at a lower risk of breast cancer relative to their non-South Asians counterparts (Winter *et al*, 1999). The findings from the present study seem to indicate that South Asian women not only have a lower risk of breast cancer but, those who develop this cancer may also have a better survival relative to non-South Asian cases. Possible reasons for the survival advantage of South Asian women in South East England include ethnic differences in the biological characteristics of the disease or the host, or in the access to, and compliance with, effective treatment regimens. There is some evidence that overweight (Zhang *et al*, 1995; Galanis *et al*, 1998) and alcohol consumption (McDonald *et al*, 2002) may be associated with poorer breast cancer survival. South Asian migrant women living in England are known to have a higher body mass index, waist – hip ratio and prevalence of obesity than European Caucasian women (Pomerleau *et al*, 1999), but their consumption of alcohol is very low (dos Santos Silva *et al*, 2002). The proportion of South Asians in South East England that live in Greater London is higher than that of non-South Asians (OPCS and GRO for Scotland, 1993). Patients in London live closer to teaching hospitals with high consultant caseloads and high usage of adjuvant therapy, factors known to influence survival (Sainsbury *et al*, 1995). Future linkage of cancer registry data with case note information on the patient and tumour characteristics as well as on referral and treatment patterns may help to estimate the contribution of each of these factors to the observed ethnic differences in survival.

## Appendix

In a relative survival model the rate (hazard) function of a cancer patient *i*, *λ_i_* (*t*), at time *t* since diagnosis, is modelled as the sum of a reference (expected) mortality rate, *λ_i_*^*^ (*t*), and an excess mortality rate (hazard), *υ_i_* (*t*), which represents the effect of the cancer diagnosis. Formally,

The parameter *λ_i_*^*^ (*t*) is not estimated but taken from published national or regional life tables. Its value depends on patients' characteristics, such as age and sex, hence it carries the suffix *i*. We used various life tables to define *λ_i_*^*^ (*t*) in order to check the robustness of the results. These were the age – sex – period-specific life tables for England and Wales as well as the equivalent ones for North and South Thames regions. We also used age – sex – period-deprivation-specific life tables for the same geographical areas. By contrast, the function *υ_i_* (*t*) (the excess mortality rate) is estimated from the data. In the formulation used in this paper, it is allowed to vary with time since diagnosis as a piecewise function of time. So, for *K* time intervals (0, *t*_1_), (*t*_1_, *t*_2_), …(*t*_*K*−1_, *t*_*K*_), the function *υ_i_* (*t*) is defined in terms of *K* parameters *α_j_*, *j*=1, 2,…,*K*, as

where *I_j_*=1 if *t* belongs to the interval (*t*_*j*−1_, *t_j_*), *I_j_*=0 otherwise. We used yearly intervals, so that each *α_j_* represents the additional mortality rate of patients diagnosed with cancer during the *j*th year after diagnosis, relative to subjects of the same sex, age and, possibly, deprivation alive in the same calendar period.

The term ‘relative survival’ used to denote model (A1) derives from the implied relation between the cumulative survival of a cancer subject and that of the equivalent reference subject. Formally,

where *S_i_*^*^ (*t*) is the cumulative expected survival probability for a reference subject of similar sex, age, etc. as the cancer patient and exp (*υ_i_* (*t*)) is the relative survival rate (RSR) at time *t*.

A simple generalisation of equation (A2) allows the function *υ_i_* (*t*) to incorporate explanatory variables, such as disease stage at diagnosis or ethnicity. The excess mortality rate can then be expressed, when including one covariate *z*, as

where exp (*β*) is interpreted as the excess rate ratio for a unit increase in the covariate *z*. This model can be easily fitted within the family of generalised linear models (McCullagh and Nelder, 1989).
